# Subdural Collection Above the Surgical Site After Endoscopic Surgery: A Case Report

**DOI:** 10.7759/cureus.67326

**Published:** 2024-08-20

**Authors:** George B Walbridge, Jonathan Paris, Rohan Sarna, Matthew Meroney, Sanjeev Kumar

**Affiliations:** 1 Emergency Medicine, Western Michigan University Homer Stryker M.D. School of Medicine, Kalamazoo, USA; 2 Physical Medicine and Rehabilitation, Private Practice, Stony Brook, USA; 3 Anesthesiology, University of Florida College of Medicine, Gainesville, USA; 4 Anesthesiology/Pain Medicine, University of Florida College of Medicine, Gainesville, USA

**Keywords:** interventional pain management, subdural collection, postoperative complication, subdural hematoma, endoscopic decompression

## Abstract

Endoscopic decompression continues to expand its therapeutic scope in alleviating chronic back pain. Endoscopic decompressions are minimally invasive and have rare complications. This case details a unique occurrence of a subdural collection following an interlaminar endoscopic laminotomy, facetectomy, lateral recess, and left L5 decompression. The subdural collection manifested above the operative site, despite the absence of dural puncture during the intervention. Subsequent to the procedure, the patient reported significant pain relief and presented for a follow-up appointment, disclosing new symptoms which included new weakness in his hamstrings and burning pain in his bilateral feet. A repeat MRI revealed a subdural collection, the etiology of which remains unclear given the intact dura during the surgical procedure. The MRI showed no new herniation and had objective improvement where his decompression took place. While previous cases have documented subdural collections primarily in association with dural puncture, this instance is distinctive in that regard. An intriguing aspect specific to endoscopic procedures is the potential for injury related to irrigation pressure. This scenario raises the hypothesis of a hematoma formation within the subdural space, possibly due to trauma to bridging vessels between the dura and arachnoid membrane. Alternatively, an unexpected increase in intra-abdominal or thoracic pressure may have led to elevated spinal vessel pressure, particularly affecting radiculomedullary veins traversing both the subdural and subarachnoid spaces. Further investigation and clinical monitoring are warranted to elucidate the precise mechanism underlying this subdural collection and its implications for postoperative management.

## Introduction

Due to advances in minimally invasive endoscopic techniques, they have become increasingly prevalent for treating lumbar spinal stenosis. Numerous studies have investigated the efficacy and safety of endoscopic decompression surgeries [1-6]. Spinal subdural collections appear to be a rare complication, with only one other case report documenting a subdural hematoma. This case will present the rare complication of a spinal subdural collection occurring after a full endoscopic interlaminar decompression.

## Case presentation

A 71-year-old male presented to the pain clinic with a history of chronic low back pain spanning several years. The pain radiated bilaterally to his lower extremities with the left-sided symptoms being worse compared to the right, extending down to the soles of his feet. He exhibited symptoms of neurogenic claudication and described radicular manifestations including numbness, cramping, and burning sensations. Physical examination revealed a positive bilateral straight leg raise test. Initial MRI findings indicated a disc bulge at the L4-5 level with moderate bilateral subarticular zone narrowing and mild bilateral neural foraminal stenosis.

In light of these findings, the patient underwent an L4-5 transforaminal epidural steroid injection, which initially provided significant pain relief for approximately two weeks, confirming the L4-5 disc bulge as the primary pain generator. The patient underwent successful interlaminar left-sided decompression at the L4-5 level, resulting in sustained pain relief.

**Figure 1:**
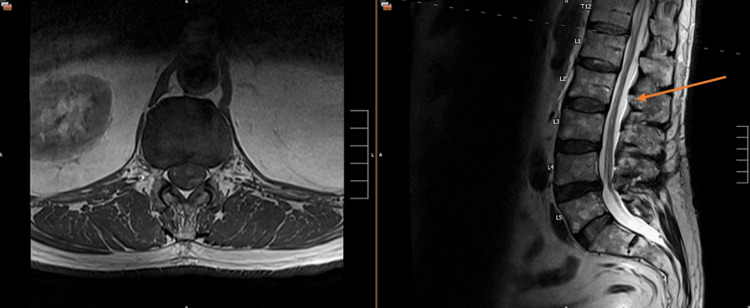
Postoperative subdural fluid collection. The arrow represents the area where the collection begins and the anterior displacement of the nerve root.

However, two weeks post-procedure, the patient experienced acute leg pain and numbness following an aggressive forward flexion bending episode, prompting a repeat MRI to evaluate for potential recurrence or new herniation. Initially suspected to have a new herniation at L4-5, the patient received a left-sided epidural injection. At a follow-up evaluation two months post-procedure, the patient continued to report radicular symptoms. The repeat MRI in Figure 1 showed objective improvement in the L4-5 disc bulge, yet noted anterior displacement and thickening of the cauda equina nerve roots. The initial radiology report suggested findings consistent with postoperative arachnoiditis, which was subsequently clarified after further discussion with the radiologist that it was in fact a fluid collection.

The MRI did show what appeared to be a subdural collection of an unknown fluid above the surgical site at the disc area from L3 to T12 as seen in Figure 1. The patient's symptoms continued to improve since the procedure, and the decision made by the patient was for observation, as he began to have increased daily function. There was a discussion to consider drainage of his symptoms to help release the pressure and allow inspecting the fluid to see if it was blood, saline, or cerebrospinal fluid (CSF).

## Discussion

Spinal subdural hematoma (SSH) is a rare but serious condition characterized by the accumulation of blood between the dura mater and the arachnoid mater of the spinal cord. It can be distinguished from a subdural hygroma, which is a fluid collection in the subdural space that is clear or only slightly xanthochromic [1]. The true incidence of SSH is difficult to ascertain due to its rarity. SSH can occur spontaneously or secondary to trauma, vascular malformations, or iatrogenic causes such as lumbar puncture, surgery, and epidural anesthesia [2-4]. 

Symptoms of SSH typically arise from spinal cord compression by the hematoma, leading to motor, sensory, and/or autonomic dysfunction [5]. Clinical presentation can vary depending on the size and location of the collection but can include severe back pain, progressive neurological deficits, as well as radicular pain [6]. When SSH is suspected, MRI is the gold standard for diagnosis [5]. 

Our case is notable because it occurred after endoscopic decompression without intraoperative dural injury or postoperative complications. While previous reports have linked SSH to surgical trauma, only one other case has reported SSH following an endoscopic procedure [7-9]. In our case, the patient reported improvement of his initial symptoms after the procedure, until postoperative day 14 when he was bending over and noticed a pop in his back. At that point, he had an acute change in his symptoms which have remained stable. This would be atypical of a SSH given this would generally present within the first few days of surgery. 

The etiology of the collection is unclear, although SSH and a collection of irrigation fluid are the most likely explanations. For the former, the mechanism could be secondary to injury of the bridging vessels between the dura and arachnoid membrane. As there was no intraoperative injury of the dura, hematoma formation may be secondary to manipulation of the dura. Interestingly, the collection was found to begin superior to the level at which the surgery was performed and spanned several levels of the spinal cord from L3 to T12. Another hypothesis is increased abdominal or thoracic pressure being transmitted to the spinal vessels, in particular the radiculomedullary veins, leading to hematoma formation.

During endoscopic surgery, high-pressure irrigation fluid is used, which could lead to the accumulation of fluid in the subdural space. The pressure from the irrigation fluid may have also contributed to injury of the subdural vessels previously mentioned. Interestingly, the patient had a delayed presentation after surgery, which lowered the suspicion for SSH.

SSH is an exceptionally rare complication of endoscopic spine surgery, but this diagnosis must be considered in patients with severe postoperative pain or new neurologic deficits. Urgent MRI evaluation is crucial to assess for fluid collections in the epidural or subdural space. If imaging reveals a subdural collection and the patient has neurologic symptoms, emergency decompression via durotomy and open evacuation may be indicated [9,10]. Otherwise, patients can be treated conservatively [11].

Notably, our case initially presented with postoperative imaging read as possible postoperative arachnoiditis due to the anterior deflection of the nerve roots, but further evaluation was more consistent with a subdural collection with arachnoiditis not being completely excluded. It is imperative to diagnose SSH early and treat it accordingly as it could exert pressure on the neural elements resulting in paralysis. 

One of the most valuable points to this case is that there is a subdural collection that was overlooked by radiology as it was hyperintense on T2 and relatively hypointense on T1 MRI which made the subdural collection hard to detect. This was only caught due to the anterior deflection of the nerve roots. This fluid collection was hyperintense compared to the CSF which could indicate an old hematoma. This highlights the fact that there is an evolution to the progression of a hematoma which can camouflage with the surrounding anatomy. Table 1 highlights the changes you would expect on an MRI of the hematoma if present.

**Table 1:** Hematoma appearance based on timing on MRI.

Time since the onset of hematoma	T1 MRI	T2 MRI
Hyperacute <1 day	Isointense	Isointense to hyperintense
Acute 1-3 days	Isointense to hypointense	T2 shortening to hypointense
Early subacute 3-7 days	T1 shortening to hyperintense	Hypointense
Late subacute 7-28 days	Hyperintense	Increases to hyperintense
Chronic >28 days	Isointense	Hyperintense

Another valuable point in this case is the patient continued to improve on his own. This leaves the possibility of a "wait-and-watch" approach to this finding after an endoscopic procedure. This could help future clinicians with similar findings and prevent unnecessary evacuations on subdural collections.

## Conclusions

This case highlights a rare complication following minimally invasive endoscopic lumbar decompression. To our knowledge, there is only one other reported case of a subdural hematoma occurring as a post-surgical complication. There have been no reported cases of an unknown subdural collection. In instances where complications arise post-surgery, imaging is essential to assess the extent of these issues. If a patient presents with severe pain or neurological deficits, open evacuation with a durotomy may be necessary. Further investigation and additional studies are required to elucidate the underlying mechanisms of this process.
